# Effectiveness of Ultrasound Imaging in Assessing the Palpation Skills of Rotating Physicians

**DOI:** 10.3389/fgene.2022.894716

**Published:** 2022-06-08

**Authors:** Peizhen Huang, Bin Zheng, Shan Liu, Lin Xu, Chengchun Chen, Shubei Zhan

**Affiliations:** ^1^ Department of Ultrasound and Imaging, Wenzhou Central Hospital, Wenzhou, China; ^2^ Wenzhou Medical University, Wenzhou, China

**Keywords:** ultrasound, palpation, joint, bone, anatomy

## Abstract

As an important means of physical examination, palpation is usually limited to the physical examination before surgery and used as an auxiliary method for disease diagnosis in the field of surgery. In practice, palpation is also used in every aspect of the surgical procedure, and its application is of great significance to surgery. The purpose of this study was to investigate the ability of ultrasound imaging to assess the ability of rotating physicians to locate musculoskeletal structures by palpation. Rotating physicians were asked to palpate and locate the long head tendon of the biceps (LHB), posterior tibialis (TPT), acromioclavicular joint (ACJ), and medial tibiofemoral joint (TFJ) spaces on two volunteer models. After positioning, a truncated steel needle was attached to the skin and parallel to the palpable structure, and the position of the steel needle relative to the designated structure was assessed by ultrasound imaging, using the Cohen kappa test to study the inter-rater agreement. The results showed that the assessor’s Kappa coefficient for judging the location of all structures was 0.816, LHB was 1.00, TPT was 0.912, ACJ gap was 0.796, and TFJ medial space was 0.844, and the success rate of palpation for TPT was 62.2%, TFJ medial space was 37.8%, ACJ clearance was 24.3%, and LHB was 8.1%. In conclusion, the teaching methods of anatomy and palpation skills need further improvement, and ultrasound imaging is an effective tool for assessing palpation skills.

## Introduction

The classic definition of palpation is “a diagnostic method in which a physician judges a disease by feeling with the hand.” In the field of surgery, it seems more appropriate to describe it as “a method of diagnosis and treatment in which a physician judges and treats diseases through the feeling of the hand.” Palpation, along with a thorough understanding of the underlying anatomy, is an important part of patient examination and diagnosis by which clinicians look for landmarks and assess musculoskeletal structures for pain, location, tone, range of motion, size, shape, temperature, and texture. Palpation can also be used to detect red flags of serious illness (Ning et al., 2019; Author Anonymous, 2014; Zhang and Sui, 2016a; Merz et al., 2013). Good palpation skills are considered a core skill for clinicians, and most studies on the accuracy of palpation skills have been conducted with experienced clinicians or have focused on the spinous processes, although spinal positioning is critical for the diagnosis and treatment of spinal pathology. It is also important to know whether the physician can accurately locate other musculoskeletal structures. According to the process of the operation, the application of palpation in operations is mainly reflected in the aspects of positioning, searching, characterization, separation, hemostasis, and evaluation (Author Anonymous, 2014; Zhang and Sui, 2016a; Snider et al., 2011; Chen et al., 2018; Xie et al., 2013; Qing et al., 2012). Palpation of musculoskeletal structures is a skill that is not easily trained, and students are often assessed for their ability to locate structures by experienced clinicians whose palpation skills are considered the gold standard. However, although palpation improves with experience, the literature shows that even among experienced clinicians, there are problems related to the reliability and accuracy of palpation (Cooperstein et al., 2015; Whittaker et al., 2019).^,^ Although ultrasound imaging provides a new tool for clinicians to assess palpation skills, few studies have used ultrasound to objectively assess the accuracy of the palpation skills of rotating physicians. Therefore, the overall goal of this study was to use ultrasound to assess the ability of rotating physicians to locate musculoskeletal structures by palpation.

## Methods and Objects

### Method

Rotating physicians who joined our hospital from July 2017 to April 2021 were included in the prospective study. This cross-sectional descriptive study was approved by the hospital ethics committee (Ethical approval K20200916), and written informed consent was obtained from all rotating physicians. Inclusion criteria include voluntary participation in the study and signed informed consent, completion of all theoretical and practical courses on musculoskeletal anatomy, i.e., lectures, anatomy lab, training in superficial anatomy, and palpation skills of musculoskeletal structures, and a clinical master’s degree; the rotation time was within one year. Exclusion criteria include non-anatomy and palpation skill courses in other training programs, i.e., kinesiology, massage therapy, and chiropractic, prior to admission to the internship, simultaneously recruiting two volunteers with similar anthropometric characteristics, pain in the anatomical area of the four structures palpated, previous surgery, and any anatomical deformity.

### Methods

**FIGURE 1:**
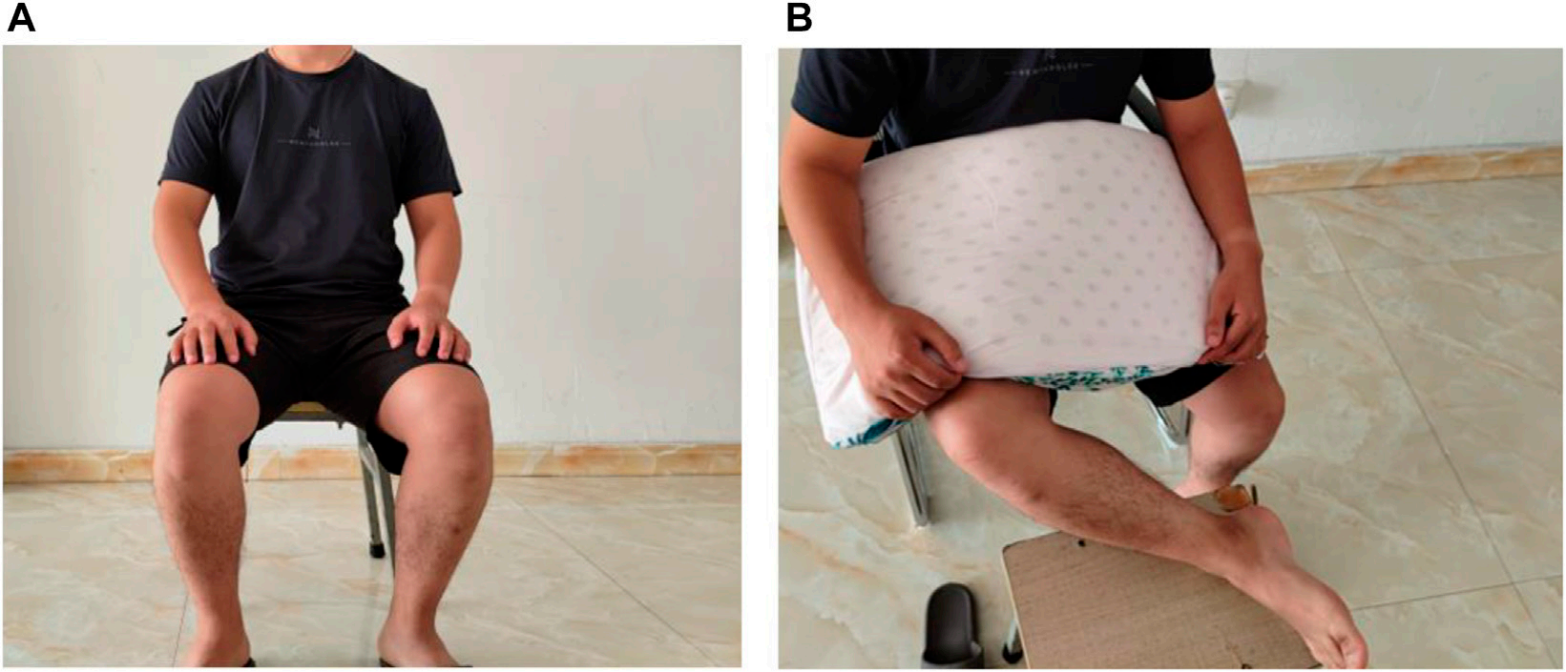
Schematic diagram of the human body model. **(A)** Positioning ACJ and TFJ in this position. **(B)** Positioning LHBT and PTT in this position.

The rotating physician was asked to locate the ACJ gap, medial TFJ gap, LHB, and TPT of the volunteer model: volunteer 1 had his back straight, knees bent at a 90° angle, his feet dangling, and his arms next to his body (Figure 1A). Volunteer 2’s lower extremity was supported on a stool in a “4” position (knees flexed at a 90° angle and heels pointed to opposite knees); the arm being assessed was next to the body (neutral position with no shoulder rotation), and the forearm was supported by a pillow, with the elbow bent at a 90° angle (Figure 1B). The rotating physician was asked to locate the ACJ gap first in volunteer 1, then the medial space in the TFJ, and then the LHB and TPT in volunteer 2. The target muscle could be instructed to resist isometric contractions to help locate the tendon, and a goniometer could be used. All joint angles were measured, adjacent structures were accessible, but no questions or assistance was allowed, and each positioning did not exceed 5 minutes.

After positioning the corresponding structure, a 3 cm long truncated steel needle (easy to be identified by ultrasound) was placed flat on the skin just above the positioning for ultrasound palpation verification. For the joint cavity, the truncated needle was placed above the joint cavity and parallel to the bone surface, and for the tendon, the truncated needle was placed on the tendon and parallel to the fibers. The needles were fixed with tape and exposed in the middle to optimize the quality of the ultrasound images. The volunteers remained stationary after the needles were fixed until the ultrasound images were captured. The ultrasound image capture was performed by a senior radiologist using ultrasound with a 13-MHz linear array transducer, and the images were saved and anonymized after capture.

**FIGURE 2:**
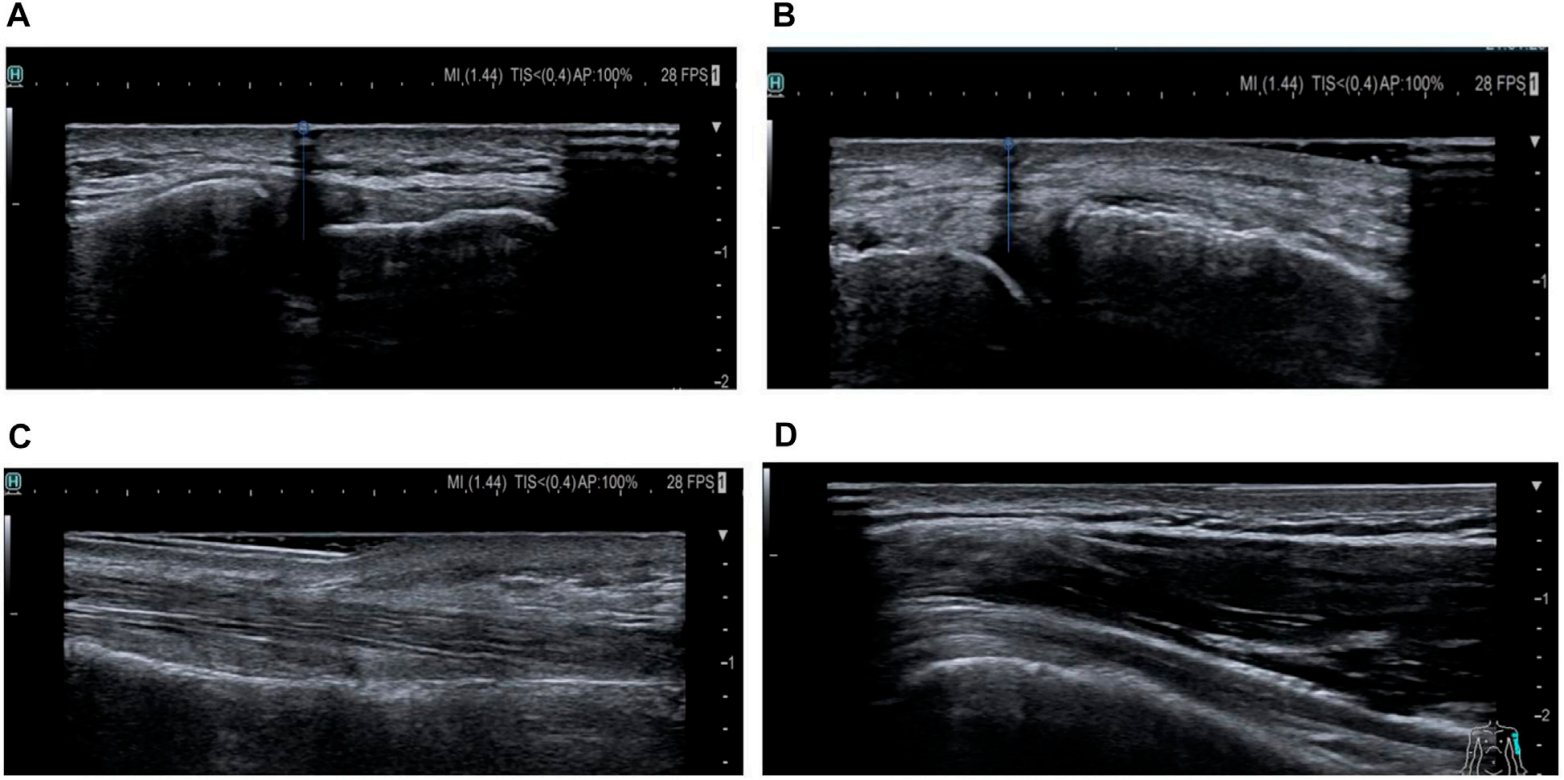
Needle placement based on target structures relative to ultrasound images. **(A)** Correct localization map of the ACJ. **(B)** Correct localization map of the TFJ. **(C)** Map of mislocalization of the long head of the biceps tendon. **(D)** Map of the mislocalization of the posterior tibialis muscle.

The main outcome was the success of the abovementioned musculoskeletal structure localization based on the target structure and the position of the steel needle on the ultrasound image. A consensus was reached after analysis by two musculoskeletal ultrasound imaging experts (a radiologist and an orthopedic surgeon). In the US image, the machine’s built-in calipers were used to draw a vertical line starting from the center of the needle and extending toward the structure. Successful palpation: the line must fall precisely between the two bones that locate the joint cavity, between the clavicle and the acromion of the ACJ (Figure 2A) and between the tibial and femoral condyles of the TFJ. If the line falls on the bone, palpate diagnosis is considered a failure, and the same principles apply to the tendon: a vertical line falling between the medial and lateral edges of the tendon is a successful localization, and a line falling outside the aforementioned medial or lateral edges is a failure to palpate (Figures 2C and D). In addition, the direction of palpation failure was assessed, that is, medial or lateral relative to the ACJ gap or LHB, relative to the TFJ medial gap, or proximal or distal to the TPT, by ultrasound measurement software to calculate the distance between the extension line and the edge of a given structure. The vertical distance was used to quantify the magnitude of the failed palpation assessment.

### Statistical Analysis

Descriptive analysis (mean, standard deviation (SD), range, counts, and percentages) was used to describe the rotating physician sample, model, and primary outcome, and after using established criteria to determine the success or failure of each palpation, the inter-rater agreement was checked using the weighted Cohen kappa coefficient, which was set as follows: ≤0: inconsistent; 0.01–0.20: very low consistency; 0.21–0.40: average consistency; 0.41–0.60: medium consistency; 0.61–0.80: high consistency; and 0.81–1.00: almost perfect.

## Results

### Characteristics of Rotating Physicians

The anthropometric characteristics of the two volunteers who volunteered as models are as follows: volunteer 1: weight = 59 kg, height = 1.65 m, and body mass index (BMI) = 21.6 kg/m^2^ and volunteer 2: weight = 60 kg, height = 1.68 m, and BMI = 21.3 kg/m^2^ (Table 1).

**TABLE 1:** Characteristics of rotating physicians.

	*n* = 37
Age (years)	23.7 ± 1.1
Male	24
Internship time (years)	0.67 ± 0.04
BMI	22.5 ± 3.2
Dominant hand (right)	33

### Inter-Rater Agreement

The consistency between the raters was checked using the weighted Cohen kappa coefficient, and the results showed that the judgment of the success palpation of the overall structure was almost completely consistent among the raters. When each structure was independently observed, it was observed that the judgment of the location of the ACJ gap in the medial space of the TPT and TFJ was highly consistent, and the judgment of the LHB was completely consistent (Table 2).

**TABLE 2:** Evaluation of palpation results by rotating physicians and Kappa analysis.

		Orthopedic physician	Positioning successful	Positioning failed	Total	Kappa
All structures		Positioning successful	41	8	49	0.816
		Positioning failed	15	84	99	
		Total	56	92	148	
TPT	Radiologist	Positioning successful	21	2	23	0.912
		Positioning failed	1	13	14	
		Total	22	15	37	
TFJ medial clearance		Positioning successful	11	3	14	0.844
		Positioning failed	2	21	23	
		Total	13	24	37	
ACJ clearance		Positioning successful	8	1	9	0.796
		Positioning failed	3	25	28	
		Total	11	26	37	
LHB		Positioning successful	3	0	3	1.000
		Positioning failed	0	34	34	
		Total	3	34	37	

### Palpation Results

Across all palpation trials, 33.1% of palpation succeeded in locating a given structure, with rotating physicians being the most successful in locating the TPT (62.2%), followed by the medial space of the TFJ (37.8%), the ACJ gap (24.3%), and finally the LHB (8.1%, Table 3). For LHB positioning, 79.4% of wrong positioning occurred in the medial direction, and the error margin in this direction was significantly larger than that in the lateral direction. For ACJ gap positioning, 64.3% of wrong positioning occurred in the medial direction, which was slightly larger than that in the lateral direction. For the medial TFJ gap localization, 73.9% of failed palpations were in the proximal end, with similar margins of error in both directions, and for TPT, failed palpation occurred in similar proportions (57.1% vs. 42.9%) in both directions, with a significantly larger margin of error in the distal direction (Table 4).

**TABLE 3:** Number of successes and failures using palpation to locate musculoskeletal structures.

	Positioning successful	Positioning failed
All structures	49	99
TPT	23	14
TFJ	14	23
ACJ	9	28
LHB	3	34

**TABLE 4:** Vertical distance between the reference line and given structure.

Structure	Offset position	Offset position	*P*
TPT	Proximal	Remote	
Positioning failed (*n* = 14)	8	6	0.529
Offset distance (mm)	4.1 ± 1.2	7.2 ± 1.8	0.032
TFJ medial gap	Proximal (femoral condyle)	Remote (tibial condyle)	
Positioning failed (*n* = 23)	17	5	0.001
Offset distance (mm)	11.4 ± 3.2	12.5 ± 3.6	
ACJ gap	Medial (clavicular)	Lateral (acromial)	
Positioning failed (*n* = 28)	18	10	0.011
Offset distance (mm)	11.6 ± 2.6	8.9 ± 2.1	0.158
LHB	Medial	Lateral	
Positioning failed (*n* = 34)	27	7	0.001
Offset distance (mm)	15.3 ± 4.6	10.8 ± 2.7	0.019

## Discussion

This study reports an inter-rater agreement on the success of assessing palpation of musculoskeletal structures in judging ultrasound imaging assessments and provides raw data on rotating physicians’ skills in locating two joint cavities and two tendons. The inter-rater agreement ranged from high agreement to perfect, and the findings support that ultrasound can be an assessment tool for assessing rotating physicians’ palpation skills for the four musculoskeletal structures targeted in this study.

Since the sample consisted of rotating physicians within one year of admission, the palpation success rate was low (33.1%). Although they had completed musculoskeletal anatomy courses during their studies, learning anatomy is an ongoing process, and their knowledge was not fully integrated with clinical practice. In addition, their anatomical concepts were mainly obtained from anatomy books, and it was difficult for them to transfer anatomical concepts learned in books to living people when individual anatomy and anthropometry changed. This population was specifically selected because of the desire to detail their palpation skills at an early stage to understand the strengths and weaknesses of rotating physicians. While this study focused on four musculoskeletal structures, the acromioclavicular joint space (ACJ), the medial space of the knee joint (TFJ), the long head of the biceps tendon (LHB), and the tibialis posterior tendon (TPT), the choices of these structures for pedagogical and clinical reasons are as follows: they are often used as landmarks and differential diagnoses, can be used to evaluate common treatments (corticosteroid injections, assisted joint mobility, and dry needling), and can be accurately visualized by US imaging (Gazzillo et al., 2011; Rho et al., 2014; Henderson et al., 2015; Walrod et al., 2018; Woods et al., 2018).

The TPT was the structure with the highest localization success rate (62.2%), confirming that students performed better in localizing joint cavities than tendons. The TPT is anatomically close to the medial malleolus, an easily located bony landmark. The medial malleolus and TPT are not obscured by the muscle mass or adipose tissue for easy palpation. There was no specific directional pattern for palpation of the affected area (proximal and distal mislocalization rates were similar); the distal direction was mostly on the flexor digitorum tendon near the TPT, and the proximal mislocalization was at the tip of the ankle. Asking volunteers to contract isometrically verifies that the structure being located is the tendon rather than bone and helps to distinguish the flexor tendon from the TPT, as demonstrated during student palpation skill training. The low success rate (8.1%) of localizing LHB in the study was between the success rate reported by Gazzillo et al. (2011) (5.3%) and the 20% success rate reported by Woods et al. (2018). The sample reported by Woods et al. consisted of clinically experienced residents, a factor that could explain the higher success rate compared to this study, and Woods confirmed that incorporating ultrasound into the palpation training curriculum increased the success rate to 51.7%, thus confirming that ultrasound-assisted teaching can have a significant adverse effect on training palpation skills. The results of this study showed that the majority of LHB mislocalization was medial, suggesting that the teaching method of LHB palpation needs to be improved, as rotation physicians can easily locate the long head of the biceps tendon closer to the medial side. The success rate of locating the ACJ gap in this study (24.3%) was close to that reported by Rho et al. (2014). A total of 10 cases of mispositioning in the rotating physicians were located on the acromion, not far from the joint space (8.9 ± 2.1 mm), indicating that students may misunderstand ACJ as the wide flat type. Similarly, Walrodd et al. (Henderson et al., 2015; Walrod et al., 2018; Woods et al., 2018) achieved a higher success rate (74.1%) in locating this structure, which could be explained by differences in standards, trainee experience, and exposure to ultrasound-assisted anatomy teaching techniques. For the TFJ medial space, the mispositioning is mostly proximal, and the distance from the joint space is 11.4 ± 3.2 mm, which means that the students are positioned to the medial femoral condyle. The mislocalization of the (ACJ) gap and the medial gap of the TFJ may indicate that it is difficult for the rotating physicians to feel the gap between the two bones, and teaching methods need to be improved to develop their recognition ability.

In conclusion, the results of this study provide data confirming that ultrasound imaging is a valuable tool in assessing the ability of rotating physicians to locate musculoskeletal structures by palpation. This study details the ability of the rotating physician to locate two joint cavities and two tendons together, although success rates for both joint cavities and the long head of the biceps tendon were low, but given the inexperience of rotating physicians in palpation skills, this is extenuating, and data on patterns of misorientation could help improve palpation skill teaching methods (Eriksson et al., 2012).

## Data Availability

The original contributions presented in the study are included in the article/Supplementary Materials, further inquiries can be directed to the corresponding author.
